# Amiodarone dose to treat ventricular tachycardia in Chagas cardiomyopathy

**DOI:** 10.1093/europace/euag142

**Published:** 2026-09-18

**Authors:** Henrique Horta Veloso, Mauro Felippe Felix Mediano

**Affiliations:** Evandro Chagas National Institute of Infectious Disease, Oswaldo Cruz Foundation, Avenida Brasil, 4.365, Rio de Janeiro, RJ 21040-360, Brazil; Evandro Chagas National Institute of Infectious Disease, Oswaldo Cruz Foundation, Avenida Brasil, 4.365, Rio de Janeiro, RJ 21040-360, Brazil

In the interesting article of Silva *et al.*,^1^ patients with Chagas cardiomyopathy (ChC), ventricular tachycardia (VT) and implanted cardioverter-defibrillator (ICD) were treated with ablation in comparison with medical therapy. They concluded that in ChC, medical therapy alone was associated with high rates of VT recurrence. However, in our opinion, the significant difference of amiodarone doses in the studied groups may be a potential confounding role for the study conclusions.

Amiodarone dose to treat VT in ChC is not established. However, the dose usually used to treat VT is higher than the low-dose usually prescribed to treat atrial fibrillation.^2^ The majority of the studies from the 1980s and 1990s investigating the role of amiodarone in ChC used doses of 400 mg/day or higher^3–6^ (*Table 1*). More recently, case series studies following patients with ChC and ICDs or clinical trials comparing patients with the electronic device to patients with medical treatment with amiodarone also used maintenance doses higher than 200 mg/day at the end of the follow-up, after eventual adjustments^7–10^ (*Table 1*). In medical practice, the differences in amiodarone dosing may reflect clinical decision-making related, for instance, to arrhythmia burden or disease severity.

**Table 1:** Studies using amiodarone to treat patients with ChC

Study	Population	Intervention	*n*	Maintenance dose (mg/day)		Follow-up
				Target dose	Final dose	
Greco *et al*.,^a^ 1980	G1: ChC with frequent PVCs G2: ChC with VT	amiodarone	G1: 34 G2: 14	G1: 50–200 G2: 400	NA	6 months
Prata *et al*.,^a^ 1982	Chronic ChC with ventricular and supraventricular arrhythmias	amiodarone	120	400–800	NA	2 months
Chiale *et al*.,^3^ 1984	Chronic ChC	amiodarone	24	600–1000	792	27 months
Vichi *et al*.,^a^ 1984	Chronic ChC with PVCs	amiodarone	20	400	NA	1 month
Haedo *et al*.,^4^ 1986	Chronic ChC	amiodarone	14	800	757	1 month
Rosenbaum *et al*.,^a^ 1987	ChC with ventricular arrhythmias	amiodarone	40	800	NA	2 months
Scanavacca *et al*.,^5^ 1990	Chronic ChC with sustained VT	amiodarone	35	400–800	356	27 months
Leite *et al*.,^6^ 2003	Chronic ChC with symptomatic VT	amiodarone	87	400	575	52 months
Cardinalli-Neto *et al*.,^7^ 2007	ChC with instable sustained VT or VF	ICD	90	300	331	25 months
Martinelli *et al*.,^8^ 2012	Chronic ChC with sustained VT or VF	ICD	90	100–600	400 (median)	45 months
Gali *et al*.,^9^ 2014	Chronic ChC with sustained VT or VF	ICD	97	300–400	NA	34 months
Martinelli-Filho *et al*.,^10^ 2024	Chronic ChC with non-sustained VT and Rassi risk score ≥10	ICD	166	100–600	277	42 months

ChC, Chagas cardiomyopathy; PVC, premature ventricular complex; VT, ventricular tachycardia; NA, not available; VF, ventricular fibrillation.

^a^Data from these studies were extracted from Stein *et al*.^2^

In the study of Silva et al,^1^ the authors carefully performed adjustments for mortality predictors which were incorporated into a multivariate analysis. Adjustments included PAINESD (Pulmonary disease, Age >60 years, Ischemic cardiomyopathy, NYHA III-IV, Ejection fraction ≤25%, electrical Storm, Diabetes) but did not include amiodarone doses. In *Table 1* of the study, the authors presented that amiodarone dose of the catheter ablation group was the double of the medical therapy group [400 mg/day (400–800) vs. 200 mg/day (100–200) respectively, *P* < 0.001].

In conclusion, differences related to arrhythmias between the studied groups may not be related only to ablation but also to the different dose of the antiarrhythmic agent. This potential limitation should be considered to analyse the results and conclusions presented.
